# Effectiveness and long-term outcomes of different crossing strategies for the endovascular treatment of iliac artery chronic Total occlusions

**DOI:** 10.1186/s12872-020-01715-7

**Published:** 2020-10-02

**Authors:** Huan Zhang, Xiangtao Li, Luyuan Niu, Yaping Feng, Xiaoyun Luo, Changming Zhang, Fuxian Zhang

**Affiliations:** Department of Vascular Surgery, Beijing Shijitan Hospital, Capital Medical University, No.10 Tieyi Rd, Haidian District, Beijing, 100038 China

**Keywords:** Chronic total occlusion, Iliac occlusive disease, Retrograde approach, Antegrade approach, Peripheral artery disease

## Abstract

**Background:**

The iliac occlusive disease is usually treated with endovascular procedures in recent years. The effectiveness of different crossing approaches for these occlusions is not precisely known. We performed a retrospective study to explore the optimal crossing approach (antegrade versus retrograde) for iliac artery chronic total occlusions (CTOs) and to examine the long-term outcomes.

**Materials and methods:**

We performed a study on 107 patients (116 iliac occlusive lesions, mean age 64.0 ± 11.1, 88 men) who underwent an iliac CTO endovascular intervention attempted with the use of both crossing strategies but were managed with one final crossing approach between August 2012 and August 2018. Baseline data, procedural characteristics, and outcomes were described. A Cox proportional hazard model and Kaplan-Meier method were developed to assess the differences in the two crossing approaches in terms of the 1-year and 5-year primary patency rates, target lesion revascularization (TLR) and major adverse limb events (MALEs).

**Results:**

Common iliac artery (CIA) lesions were more likely to be crossed successfully in the retrograde direction (6.8% for antegrade vs. 20.9% for retrograde, *p* = 0.005), while lesions in the CIA/ external iliac artery (EIA) were more prone to be crossed successfully in the antegrade direction (58.9% for antegrade vs. 39.5% for retrograde, *p* = 0.016). There were no significant differences in the crossing approach for EIA lesions between the two groups. The two crossing approaches were associated with similar estimates of 1- and 5-year primary patency, TLR and MALE rates.

**Conclusion:**

The antegrade approach was associated with a higher rate of successful crossing in CIA/EIA CTO lesions, while the CIA-only CTOs were more likely to be crossed successfully with the retrograde approach.

## Background

Lower extremity peripheral artery disease (PAD) is common and affects millions of people worldwide. CTOs of the iliac arteries account for 20–40% of lesions in patients who undergo treatment for symptomatic PAD [1]. In the last decade, open surgery has been the standard method for severe occlusive lesions, but recent advances in interventional techniques and devices allow percutaneous revascularization and present comparable patency rates during long follow-up for patients with iliac occlusive disease regardless of lesion complexity. As a result, this endovascular-first concept has been a regular concept in the majority of institutions worldwide [2, 3].

Guidewire crossing, whether intraluminal or subintimal, is the first and most difficult step before treatment for CTO. Some authors recommend a retrograde crossing approach for CIA occlusions and an antegrade crossing approach for EIA occlusions, while the research on this topic is limited [4]. Kokkinidis et al. reviewed the patients who received iliac artery angioplasty to analyze the outcomes of two crossing approaches, but some patients were treated successfully with only one approach, which might have a bias on the efficacy of the crossing approach [5]. In this study, we collected data of iliac CTO patients who had undergone endovascular treatment in our center and compared their immediate and long-term outcomes with antegrade crossing versus retrograde crossing approaches.

## Methods

This retrospective study using data derived from our hospital was approved by the institutional review board and was in accordance with the Declaration of Helsinki.

### Study population and data collection

One vascular surgeon reviewed all patients with iliac CTOs who underwent endovascular therapy between August 2012 and August 2018. Data of patients who underwent endovascular treatment for iliac lesions which were attempted with both the antegrade and retrograde approaches and were successfully crossed were collected based on the procedure notes. The exclusion criteria included the presence of a concomitant aorta/iliac aneurysm or dissection, previous stent implantation, or transfer to open surgery. Baseline demographic, clinical, laboratory, and procedure data as well as lesion characteristics and outcomes were obtained from electronic medical records, angiograms, and telephone follow-ups.

Patients and lesions were characterized based on the use of an antegrade or a retrograde final crossing approach during the interventional procedure. When bilateral occlusive iliac arteries were revascularized endovascularly during the same procedure, they were counted as two different lesions. However, ipsilateral CIA and EIA CTOs were counted as one combined lesion (CIA/EIA).

### Endovascular procedure

All patients provided written informed consent and were administered aspirin (100 mg) and clopidogrel (75 mg) 3 days before the procedure. For these complex lesions, arterial access options included the ipsilateral common femoral artery (CFA) combined with the contralateral CFA, the ipsilateral CFA combined with the brachial artery, and a combination of all three. The choice of approach was up to the experience of the operator.

**Fig. 1:**
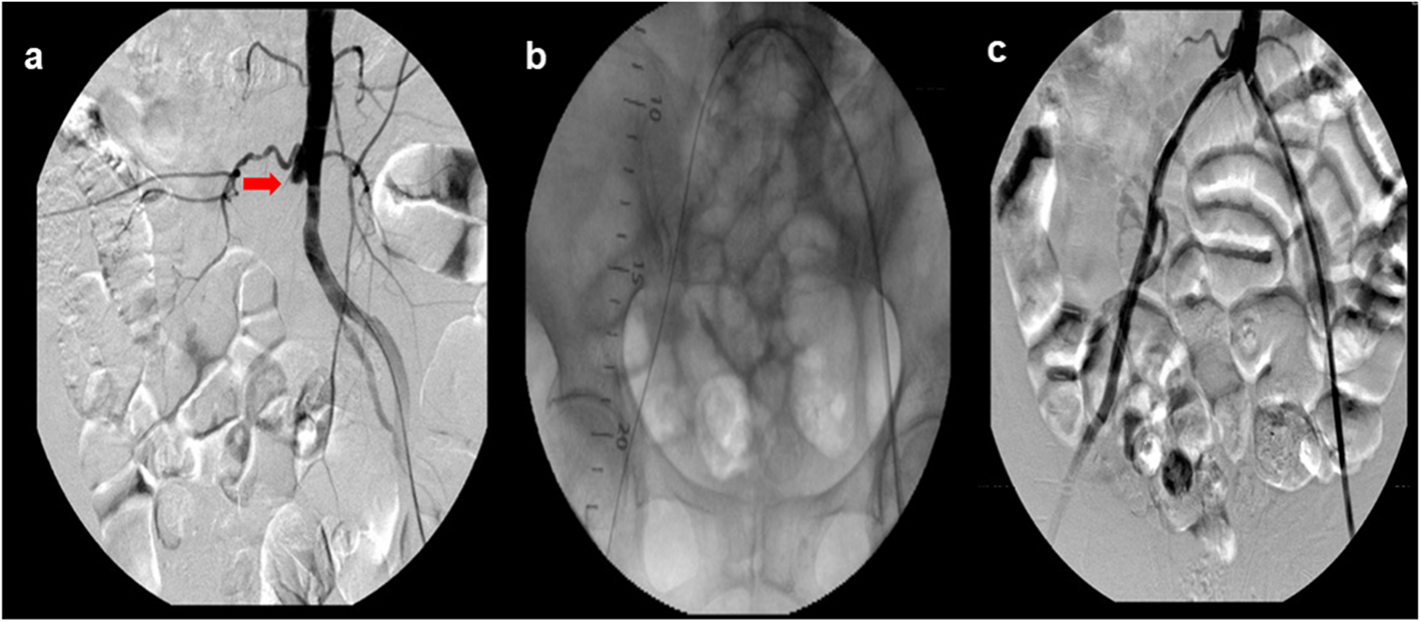
**a**, angiography showing a stump at the proximal end of right common iliac artery and external iliac artery occlusion (arrow); **b**, successfully antegrade crossing the lesion from contralateral side after the failure of retrograde approach; c, excellent result with stent implantation

CIA occlusion was more likely to be treated with a retrograde crossing approach initially in our center, while endovascular treatment for EIA lesions was attempted using the antegrade approach. Additionally, the retrograde approach was the first choice for occlusions of both the CIA and EIA. Whenever one single access was hard to pass through the occlusion, combined accesses from two directions (antegrade and retrograde) were performed (Fig. 1). Normally, vascular accesses were established under ultrasound guidance, but an incision was chosen if endarterectomy was performed for severe femoral artery atherosclerosis. Antegrade and retrograde revascularization approaches were attempted with different kinds of hydrophilic guidewires. When the guidewire failed to pass through the CTO lesion in one direction, the other direction was considered. Passing through only part of the subintimal space was acceptable. If the guidewire was not able to re-enter the true lumen in two directions, re-entry techniques may be needed. Because re-entry devices were not available in our center, alternative measures including the controlled antegrade and retrograde tracking and dissection (CART) technique, rendezvous technique, and bidirectional balloon angioplasty technique were the typical options. Gradual balloon angioplasty and stent implantation were performed following successful final guidewire crossing. Sometimes, the flossing technique (with or without a snare) was required to guarantee the secure passage of angioplasty and stenting, which would facilitate the subintimal recanalization of CTOs. Under the circumstance of bilateral proximal CIA lesions or those combined with distal aortic lesions, kissing stenting was considered for reconstructing the aortic bifurcation. After the procedure, the puncture points were closed with ProGlide (Abbott, USA) or compressed manually until hemostasis. Every patient without an obvious risk of hemorrhage was administered two antiplatelets or one antiplatelet plus rivaroxaban (10 mg qd) for at least three months, followed by one antiplatelet for life.

### Definitions and outcomes

Lesion success was defined as final crossing and restored vessel patency with residual stenosis < 30%, while procedure success was defined as lesion success without significant complications (dissection, perforation, or embolism). Lesion patency was determined by a peak systolic velocity ratio < 2.5 on duplex ultrasound. Claudication was classified as Rutherford categories 1 to 3, and critical limb ischemia (CLI) was classified as Rutherford categories 4 to 6. All lesions were categorized according to the TransAtlantic Inter-Society Consensus II (TASC) classification. Major adverse limb events (MALEs) were defined as target lesion bypass, thrombolysis, or target limb major amputation [6].

The primary outcomes of the study were the primary patency rates at 1 and 5 years of follow-up. The secondary outcomes included 1- and 5-year target lesion revascularization (TLR), MALE rates, and intraprocedural complications. For all the patients’ outcomes, the comparison of those whose lesions were finally crossed with an antegrade approach versus a retrograde approach was conducted.

### Statistical analysis

Continuous variables were presented as mean ± standard deviation and categorical data as frequencies and percentages. Data were tested for normality using the Shapiro-Wilk normality test. Non-normal distribution data were compared with non-parametric tests while Fisher exact and chi-square tests were used for categorical data. The Kaplan-Meier method was constructed to estimate primary patency rate and freedom from MALE in 116 lesions (73 antegrade and 43 retrograde). A univariate Cox proportional hazard model was developed to assess the relationship between baseline variables and 1-year and 5-year Primary patency rate, TLR and MALE; Hazard ratios (*HRs*) were provided with 95% confidence intervals (*CIs*). The estimates were compared with the log-rank test and Kaplan-Meier curves. A logistic regression model was established to identify the association between baseline variables and subintimal crossing. Then variables that had either a statistically significant correlation in the univariate analysis (*p* < 0.05) or probably had a significant clinical association were assessed in a multivariate logistic regression model; results of logistic regression are given as the odds ratio (*OR*) with the 95% CI. For all data, *p* < 0.05 was recognized as statistically significant, and the SPSS version 25.0 (IBM Corp., Armonk, NY, USA) was used for statistical analysis.

## Results

### Patient characteristics

During the study period, 207 patients underwent endovascular treatment for iliac artery occlusions in our medical center. Based on the exclusion and inclusion criteria, 107 patients (116 iliac CTO lesions) were included in the two study groups. Every CTO lesion crossing was attempted with two approaches and was finally accomplished in the antegrade or retrograde direction. Sixty-seven patients (73 iliac CTO lesions) were included in the antegrade crossing group, while 40 patients (43 iliac CTO lesions) were included in the retrograde crossing group. The baseline data of all the patients are presented in Table 1. Based on the ankle brachial index (ABI) value, occlusions of patients in the antegrade crossing group were more severe than those of patients in the retrograde crossing group (0.25 ± 0.21 vs. 0.29 ± 0.22, *p* = 0.005). There were no significant differences in the other baseline characteristics or in comorbidities between the two groups. According to the Rutherford category, all the patients were in grade 3 or more, and 75.8% in grade 4–6. The percentage of patients with grade 4–6 was similar between the two groups [57 (78.1%) vs. 31 (72.1%); *p* = 0.446].

**Table 1:** Baseline characteristics

Variables	Total	Antegrade (*n* = 73)	Retrograde (*n* = 43)	*P*-value
Age, mean (SD)	64.03 ± 11.1	64.04 ± 10.7	64.02 ± 12.78	0.862
Male, *n* (%)	88 (75.8)	55 (82.1)	33 (82.5)	0.864
Stroke history, *n* (%)	28 (24.1)	21 (28.7)	9 (20.3)	0.352
MI history, *n* (%)	22 (18.9)	12 (16.4)	8 (18.6)	0.766
Diabetes, *n* (%)	37 (31.8)	24 (32.8)	10 (23.3)	0.273
CAD, *n* (%)	49 (42.2)	28 (38.4)	21 (48.8)	0.273
Hypertension, *n* (%)	74 (63.7)	50 (68.5)	24 (55.8)	0.171
Dyslipidemia, *n* (%)	47 (40.5)	31 (42.5)	16 (37.2)	0.578
CHF, *n* (%)	13 (11.2)	8 (11.0)	5 (11.6)	0.912
Smoking, *n* (%)	42 (36.2)	29 (39.7)	12 (27.9)	0.198
EGFR, mean (SD)	83.6 ± 13.02	84.55 ± 13.0	81.99 ± 13.07	0.793
CLI, *n* (%)	69 (59.4)	45 (61.6)	24 (55.8)	0.537
ALI, *n* (%)	19 (16.3)	12 (16.4)	7 (16.3)	0.982
Rutherford 3, *n* (%)	28 (24.1)	16 (21.9)	12 (27.9)	0.446
Rutherford 4–6, *n* (%)	88 (75.8)	57 (78.1)	31 (72.1)	0.446
ABI, mean (SD)	0.27 ± 0.21	0.25 ± 0.21	0.29 ± 0.22	0.005
Aspirin (*n*)	57 (49.1)	38 (52.1)	19 (44.2)	0.413
Clopidogrel (*n*)	22 (18.9)	17 (23.3)	5 (11.6)	0.122
Statin (*n*)	37 (31.8)	23 (31.5)	14 (32.6)	0.907

*ABI* Ankle-brachial index, *ALI* acute limb ischemia, *CAD* coronary artery disease, *CHF* congestive heart failure, *CLI* chronic limb ischemia, *EGFR* estimated glomerular filtration rate, *MI* myocardial infarction, *SD* standard deviation

### Lesion characteristics and therapeutic procedures

The lesion and therapeutic characteristics are presented in Table 2. There were no significant differences in intraprocedural complications (perforation: 0 for antegrade vs. 2.3% for retrograde; dissection: 1.3% for antegrade vs. 2.3% for retrograde; and embolism: 2.7% for antegrade vs. 0 for retrograde; *p* > 0.05). Only one case of perforation, which was located at the external iliac artery and led to hypovolemic shock, was excluded with a covered stent. CTO lesions crossed with the antegrade approach were more severe in terms of the TASC categories and were longer than those crossed with the retrograde approach (TASC D: 74.0% for antegrade vs. 51.2% for retrograde, *p* = 0.013; length: 8.93 ± 2.37 for antegrade vs. 5.58 ± 2.28 for retrograde, *p* = 0.021). CIA lesions were more likely to be crossed successfully in the retrograde direction (6.8% for antegrade vs. 20.9% for retrograde, *p* = 0.005), while CIA and EIA lesions were more likely to be crossed successfully in the antegrade direction (58.9% for antegrade vs. 39.5% for retrograde, *p* = 0.016). There were no significant differences in the crossing approach for EIA lesions between the two groups. Nearly half of the lesions were treated with subintimal crossing in each group; however, the retrograde approach was more likely to pass through the subintima and more likely to require a re-entry technique, such as the “bidirectional balloon angioplasty technique” (subintimal: 45.2% for antegrade vs. 67.4% for retrograde, *p* = 0.02; re-entry: 11.0% for antegrade vs. 34.9% for retrograde, *p* = 0.002). In the antegrade group, the brachial artery was more likely to be used for those with ostium occlusion of the common iliac artery (72% vs. 27%, *p* = 0.01). All three vascular accesses were prepared in 47 and 43% of patients in the antegrade group and retrograde group, respectively. There were two patients developing pseudoaneurysm at the puncture site in each group.

**Table 2:** Angiographic and procedural characteristics

Variables	Total (*n* = 116)	Antegrade (*n* = 73)	Retrograde (*n* = 43)	*P* value
CIA, *n* (%)	14 (12.1)	5 (6.8)	9 (20.9)	0.005
CIA/EIA, *n* (%)	60 (51.7)	43 (58.9)	17 (39.5)	0.016
EIA, *n* (%)	42 (36.2)	25 (34.2)	17 (39.5)	0.561
Procedural success, *n* (%)	113 (97.4)	71 (97.3)	42 (97.3)	0.891
No/mild calcification, *n* (%)	25 (21.5)	17 (23.2)	8 (18.6)	0.780
Moderate/sever calcification, *n* (%)	91 (78.4)	56 (76.7)	35 (81.3)	0.531
Target lesion length, mean (SD)	7.69 ± 2.84	8.93 ± 2.37	5.58 ± 2.28	0.021
TASC C, *n* (%)	40 (34.4)	19 (26.0)	21 (48.8)	0.013
TASC D, *n* (%)	76 (65.5)	54 (74.0)	22 (51.2)	0.013
Leichter, *n* (%)	33 (28.4)	23 (31.5)	10 (23.3)	0.341
Self-expanding stents, *n* (%)	110 (94.8)	71 (97.3)	39 (90.7)	0.123
Covered stents, *n* (%)	52 (44.8)	34 (46.6)	18 (41.9)	0.620
Kissing stents, *n* (%)	12 (10.3)	7 (9.6)	5 (11.6)	0.728
Target lesion complications, *n* (%)	5 (4.3)	3 (4.1)	2 (4.6)	0.270
Perforation	1 (0.8)	0	1 (2.3)	0.191
Dissection	2 (1.7)	1 (1.3)	1 (2.3)	0.110
Embolism	2 (3.4)	2 (2.7)	0	0.274
Atherectomy, *n* (%)	20 (17.2)	10 (13.7)	10 (23.3)	0.188
Re-entry techniques, *n* (%)	23 (19.8)	8 (11.0)	15 (34.9)	0.002
Fluoroscopy time, min	33.6 ± 12.5	35.48 ± 12.5	30.35 ± 11.87	0.509
Subintimal angioplasty	62 (53.4)	33 (45.2)	29 (67.4)	0.021
pseudoaneurysm	4 (3.4)	2 (2.7)	2 (4.6)	0.120

*CIA* common iliac artery, *EIA* external iliac artery, *SD* standard deviation, *TASC* TransAtlantic Inter-Society Consensus II

In the univariate logistic regression analysis (Table 3), calcification (*OR* 3.17, 95% CI 1.01–7.69, *p* = 0.03), lesion length > 80 mm (*OR* 4.23, 95% CI 1.66–9.45, *p* = 0.01) and smoking (*OR* 2.89, 95% CI 1.03–8.07, *p* = 0.04) were associated with a higher risk of subintimal angioplasty, while final antegrade crossing was probably associated with a lower risk of subintimal angioplasty (*OR* 0.22, 95% CI 0.05–0.52, *p* = 0.07). Nevertheless, smoking (*OR* 2.12, 95% CI 0.56–7.63, *p* = 0.13) was not included in the list, and final antegrade crossing (*OR* 0.34, 95% CI 0.14–0.84, *p* = 0.02) was significantly associated with a lower risk of subintimal angioplasty after ruling out confounding factors in multivariate logistic regression analysis.

**Table 3:** Univariate and Multivariate Logistic Regression Analysis for Association Between Baseline Factors and Subintimal Crossing Approach

Variables	Univariate	Multivariate
Calcification	3.17 (1.01–7.69) *P* = 0.03	4.27 (1.19–15.34) *P* = 0.02
Length > 80 mm	4.23 (1.66–9.45) *P* = 0.01	2.96 (1.26–6.97) *P* = 0.01
Final antegrade	0.22 (0.05–0.52) *P* = 0.07	0.34 (0.14–0.84) *P* = 0.02
Smoking	2.89 (1.03–8.07) *P* = 0.04	2.12 (0.56–7.63) *P* = 0.10
TASC	1.11 (0.49–2.51) *P* = 0.79	
ALI	1.59 (0.48–5.25) *P* = 0.44	
CLI	1.78 (0.69–4.60) *P* = 0.22	
CIA	1.29 (0.39–4.31) *P* = 0.66	
EIA	1.86 (0.26–13.10) *P* = 0.53	
Sex	1.36 (0.39–4.68) *P* = 0.62	
Rutherford	0.92 (0.13–6.36) *P* = 0.17	
Diabetes	2.20 (0.75–6.42) *P* = 0.14	
Hypertension	1.13 (0.38–3.33) *P* = 0.81	
Dyslipidemia	0.75 (0.14–3.98) *P* = 0.73	
Stroke history	2.45 (0.76–7.91) *P* = 0.13	
MI history	0.27 (0.04–1.74) *P* = 0.17	
CHF	1.27 (0.13–12.15) *P* = 0.83	
CAD	0.93 (0.29–2.94) *P* = 0.91	
Age	0.96 (0.91–1.01) *P* = 0.13	
ABI	0.97 (0.09–10.22) *P* = 0.98	
Aspirin	1.10 (0.33–3.60) *P* = 0.87	
Clopidogrel	0.80 (0.18–3.57) *P* = 0.77	
Statin	0.64 (0.10–3.99) *P* = 0.63	

*ABI* ankle-brachial index, *ALI* acute limb ischemia, *CAD* coronary artery disease, *CHF* congestive heart failure, *CIA* common iliac artery, *CLI* chronic limb ischemia, *EGFR* estimated glomerular filtration rate, *EIA* external iliac artery, *MI* myocardial infarction, *TASC* TransAtlantic Inter-Society Consensus II

### Treatment results

**Fig. 2:**
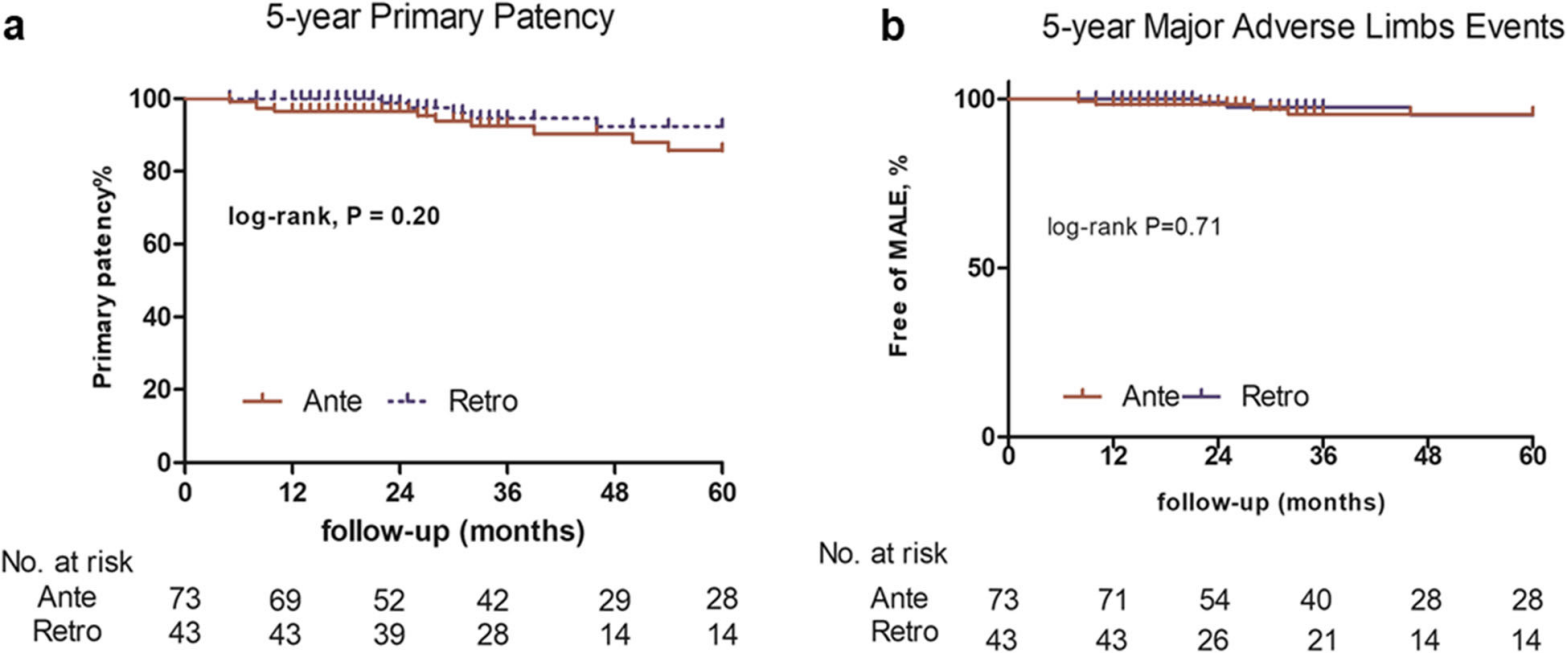
Kaplan-Meier curves of 5-year **a** primary patency and **b** major adverse limb events (MALE). There are no significant differences between these two crossing approaches

The 1- and 5-year primary patency rates for lesions treated with antegrade crossing versus retrograde crossing were 94.7% vs 97.7% (*p* = 0.44) and 79.6 vs 80.5% (*p* = 0.84), respectively. The 1- and 5-year MALE rates for lesions treated with antegrade crossing versus retrograde crossing were 2.7% vs 0 (*p* = 0.27) and 6.8% vs 11.6% (*p* = 0.69), respectively. At the same time, the 1- and 5-year TLR rates for lesions treated with antegrade crossing versus retrograde crossing were 2.7% vs 0 (*p* = 0.27) and 10.9% vs 11.6% (*p* = 0.9), respectively. During the 5-year follow-up, there were 2 (2.7%) cases of limb loss in the antegrade group and 0 in the retrograde group (*p* = 0.27). Kaplan-Meier graphs for primary patency and freedom from MALEs at up to 5 years are demonstrated in Fig. 2. Cox-regression analysis showed no difference in the 5-year rates of primary patency (*HR*: 0.97; 95% CI: 0.32–2.92; *p* = 0.84) or MALEs (*HR*: 1.35; 95% CI: 0.30–6.05; *p* = 0.69). There were no significant predictors of long-term outcomes.

## Discussion

The treatment for complex iliac occlusions has switched from open surgery to endovascular-first approaches [7]. In addition, multivessel accesses are often required during the intraluminal procedure, but the superiority of the crossing direction (antegrade vs. retrograde) is still unknown. Considering the pushability of catheters and the working efficiency, the antegrade approach is empirically preferred for EIA lesions, while the retrograde approach is preferred for CIA CTOs. However, antegrade accesses from the contralateral femoral artery or left brachial artery are also available for CIA lesions. After failure of the initial retrograde crossing approach in 99 patients with aortoiliac CTOs, Miion et al. chose the antegrade approach from the brachial artery and maintained a high technical success rate of 93% [7].

Previous studies have shown no connection between the crossing strategy and lesion position. For example, Kokkinidis DG et al. retrospectively analyzed 188 lesions and failed to find any association except for the EIA with antegrade crossing [5]. However, most of their patients underwent successful crossing with the initial attempt, so it is unknown whether the other crossing approach was available for those lesions. The patient who was successfully treated with the retrograde crossing approach might be accomplished with the antegrade approach as well. In our study, we focused on lesions with an unsuccessful initial crossing strategy and those that were managed by the final approach, which more clearly demonstrates the optimal crossing strategy. Finally, our study showed that the final crossing direction was not associated with EIA CTOs; in contrast, CIA CTOs were more likely to be crossed successfully with the retrograde approach. In addition, antegrade crossing was more efficient than retrograde crossing for CIA and EIA CTO lesions.

In this study, two or three accesses were used to cross the CTO lesions. The brachial approach had the advantages of allowing a direct line for recanalization and increasing the torque force that could be applied to guidewires. As a result, the transbrachial approach was considered when contralateral crossover failed or was not appropriate due to the anatomy of the lesions. For example, brachial access would increase the probability of crossing for a flush iliac artery occlusion, which is a CTO lesion without a stump in the origin of the CIA. The guidewire and catheter approaches from the contralateral femoral artery are prone to slipping in the aorta because of the lack of required support [4, 8]. As shown in our study, the presence of a stump in the CTO lesions was associated with the use of contralateral access (72% vs. 27%). Retrograde crossing is the preferred strategy used by some operators, as it is straight, short, and easy to manipulate, yet it has the disadvantage that it usually involves advancing into the subintimal space, which is a difficult issue for perforating thickened aortic intima and causes serious complications owing to aortic dissection. Therefore, the subintimal approach is supposed to prevent dissection from extending to the level of the aortic bifurcation, and re-entry techniques are needed [9].

As noted in the literature, anatomic characteristics may be related to crossing approaches. Severe calcification, thrombosis, and rigid plaques are hindrances for the advancement of guidewires in the true lumen [10, 11]. Statistically, we found that longer lesions with severe TASC categories were more likely to be crossed with the antegrade approach. Furthermore, the antegrade crossing approach was associated with fewer subintimal crossing and re-entry techniques. According to our data, multivariate logistic regression demonstrated that subintimal crossing was associated with severe calcifications, the retrograde approach, and long lesions.

Primary stents were deployed in all our patients. Primary stenting is preferred for preventing the arterial wall from retracting, and it can obtain an adequate patency rate, as nearly half of all CTO lesions cross through the subintimal space [12, 13]. In recent years, the majority of our stents have been self-expandable because balloon-expandable stents are not always available in our center. Usually, self-expandable stents are more flexible and are preferred for tortuous iliac arteries and for kissing stenting of the aortic bifurcation [14]. On the other hand, balloon-mounted stents are deployed for lesions that require a strong supporting force and a strictly precise placement. To prevent thromboembolism and artery rupture, covered stents may be suitable [15]. However, there is no clinical evidence of a significant advantage of one type of stent over the other based on long-term outcomes. The choice of stent depends not only on the lesion to be treated but also on the size of the introducing sheath as well as the familiarity with and availability of specific devices.

According to the TASC II guidelines, open surgery remains the gold standard of treatment for complex aortoiliac disease because it has excellent short- and long-term patency rates, which are as high as 85–92% [11, 16, 17]. The primary 2-year patency rate for complex and extensive lesions treated with endovascular procedures was 94% in one multicenter study, which is comparable to the outcomes of TASC A and B lesions [18]. Even the long-term outcomes of stent placement in complex aortoiliac CTOs are acceptable, such as the 5-year patency rate of 88% found in the study conducted by Ichihashi et al. [19]. In our retrospective study, the 1- and 5-year primary patency rates were 97.3 and 80.1%, respectively. Once crossed successfully, there were no significant differences in the long-term patency rates according to the crossing strategy.

Our retrospective study should be explicated within the context of some limitations. First, due to its retrospective nature, selection biases are unavoidable. Second, the data, such as the technical success rate, patency rate, and complication rate, were not consistent with the general outcomes of other research because our study focused only on t those iliac artery CTO lesions which were attempted with two crossing approaches. Third, the choice of vascular access depends not only on the anatomic characteristics but also on the experience of the operator. As a result, further prospective studies should be designed to explore the optimal crossing strategy for complex iliac artery CTOs.

## Conclusion

The antegrade crossing approach was more efficient for CIA and EIA CTO lesions, while the CIA-only CTOs were more likely to be crossed successfully with the retrograde approach. Furthermore, the advancing guidewire in the retrograde approach was more likely to cross the lesion through the subintimal space and was associated with a greater probability of re-entry techniques compared with that in the antegrade approach. Finally, subintimal crossing was associated with severe calcifications, the retrograde approach, and long lesions.

## Data Availability

The datasets used and/or analysed during the current study are available from the corresponding author on reasonable request.

## Abbreviations

CTOs: Chronic total occlusions
TLR: Target lesion revascularization
MALEs: Major adverse limb events
CIA: Common iliac artery
EIA: External iliac artery
PAD: Peripheral artery disease
CFA: Common femoral artery
CART: Controlled antegrade and retrograde tracking
TASC: TransAtlantic inter-society consensus II classification
HRs: Hazard ratios
CIs: Confidence intervals
OR: Odds ratio
ABI: Ankle brachial index
